# Extracellular Matrix Mimicking Nanofibrous Scaffolds Modified With Mesenchymal Stem Cell-Derived Extracellular Vesicles for Improved Vascularization

**DOI:** 10.3389/fbioe.2020.00633

**Published:** 2020-06-25

**Authors:** Dake Hao, Hila Shimshi Swindell, Lalithasri Ramasubramanian, Ruiwu Liu, Kit S. Lam, Diana L. Farmer, Aijun Wang

**Affiliations:** ^1^Department of Surgery, School of Medicine, University of California, Davis, Sacramento, CA, United States; ^2^Institute for Pediatric Regenerative Medicine, Shriners Hospitals for Children, Sacramento, CA, United States; ^3^Department of Biochemistry and Molecular Medicine, School of Medicine, University of California, Davis, Sacramento, CA, United States; ^4^Department of Biomedical Engineering, University of California, Davis, Davis, CA, United States

**Keywords:** electrospun nanofibrous scaffold, mesenchymal stem cell, extracellular vesicle, integrin-based ligand, vascularization, tissue regeneration

## Abstract

The network structure and biological components of natural extracellular matrix (ECM) are indispensable for promoting tissue regeneration. Electrospun nanofibrous scaffolds have been widely used in regenerative medicine to provide structural support for cell growth and tissue regeneration due to their natural ECM mimicking architecture, however, they lack biological functions. Extracellular vesicles (EVs) are potent vehicles of intercellular communication due to their ability to transfer RNAs, proteins, and lipids, thereby mediating significant biological functions in different biological systems. Matrix-bound nanovesicles (MBVs) are identified as an integral and functional component of ECM bioscaffolds mediating significant regenerative functions. Therefore, to engineer EVs modified electrospun scaffolds, mimicking the structure of the natural EV-ECM complex and the physiological interactions between the ECM and EVs, will be attractive and promising in tissue regeneration. Previously, using one-bead one-compound (OBOC) combinatorial technology, we identified LLP2A, an integrin α4β1 ligand, which had a strong binding to human placenta-derived mesenchymal stem cells (PMSCs). In this study, we isolated PMSCs derived EVs (PMSC-EVs) and demonstrated they expressed integrin α4β1 and could improve endothelial cell (EC) migration and vascular sprouting in an *ex vivo* rat aortic ring assay. LLP2A treated culture surface significantly improved PMSC-EV attachment, and the PMSC-EV treated culture surface significantly enhanced the expression of angiogenic genes and suppressed apoptotic activity. We then developed an approach to enable “Click chemistry” to immobilize LLP2A onto the surface of electrospun scaffolds as a linker to immobilize PMSC-EVs onto the scaffold. The PMSC-EV modified electrospun scaffolds significantly promoted EC survival and angiogenic gene expression, such as KDR and TIE2, and suppressed the expression of apoptotic markers, such as caspase 9 and caspase 3. Thus, PMSC-EVs hold promising potential to functionalize biomaterial constructs and improve the vascularization and regenerative potential. The EVs modified biomaterial scaffolds can be widely used for different tissue engineering applications.

## Introduction

Natural extracellular space is a dynamic and responsive environment consisting of non-cellular components such as soluble factors, non-soluble extracellular matrix (ECM), and extracellular vesicles (EVs) (Mathivanan, 2017). The ECM regulates many important processes including cellular proliferation, adhesion, migration, differentiation, tissue homeostasis and remodeling. These functions do not only depend on ECM's three-dimensional network, but also on its biological components (Huleihel et al., 2016). Electrospinning is a powerful technology to manufacture nano/microfibrous scaffolds that imitate the natural ECM architecture for allowing the integration of the scaffolds with surrounding cells and promoting tissue regeneration (Sarkar et al., 2006; Gao et al., 2018). We have successfully constructed electrospun scaffolds for various tissue regeneration applications, such as vascular tissue regeneration (Yu et al., 2012; Hao et al., 2020a), wound healing (Lee et al., 2012), peripheral nerve regeneration (Wang et al., 2011; Zhu et al., 2011), spinal cord regeneration (Zhu et al., 2010; Saadai et al., 2011; Downing et al., 2012) and drug delivery (Qi et al., 2006). However, the electrospun scaffolds lack biological motifs that are also included in the natural ECM and can mediate biological signaling and intercellular communication (Lu et al., 2011; Demircan et al., 2014). Surface modification plays a key role in regulating the biological interactions between cell/tissue and biomaterials (Wang et al., 2003). The functionalities of biomaterials, such as biocompatibility, adhesion and biological signaling, can be further improved by surface modification with bioactive motifs (Jiao and Cui, 2007; de Mel et al., 2012). Our previous studies have successfully improved the biological functions of electrospun scaffolds for different types of stem cells by surface modification (Hao et al., 2017, 2020b). Thus, surface modification is essential for the new functional biomaterial development and innovative medical devices design, which are contributive for advancing biomaterials in tissue regeneration and clinical applications (Wu et al., 2012; Bose et al., 2018). Vascularization is crucial for tissue development, maintenance and regeneration by to supplying nutrients and oxygen for cells and tissue (Santos and Reis, 2010; Muangsanit et al., 2018). Thus, many different approaches have been used to functionalize the electrospun scaffolds for enhancing vascularization, such as growth factors (Zhao et al., 2016; Janse van Rensburg et al., 2017), functional molecules (Lee et al., 2015; Garcia and Garcia, 2016), DNA (Scharnweber et al., 2018) and so on. In our previous studies, we demonstrated that an integrin-based ligand modified electrospun scaffold improved EC functions *in vitro* and vascularization *in vivo* (Hao et al., 2017, 2020a). However, these approaches only improve the cell and tissue functions by promoting cell/tissue-biomaterial interaction, but do not promote the biological information and substance transfer simulating the dynamic native ECM (Teodori et al., 2014; Sood et al., 2019). Therefore, to construct biofunctional scaffolds with biological information exchange and transmission will further promote the applications of biological materials in tissue regeneration.

EVs are produced in the endosomal compartment of most eukaryotic cells (Yanez-Mo et al., 2015; van Niel et al., 2018), enriched with various molecular constituents of their original cell, including lipids, proteins and RNAs, and are capable of transferring cell-to-cell signaling (van der Pol et al., 2012; Dhondt et al., 2016). Therefore, EVs have been widely used in tissue engineering area due to their multiple functions, such as pro-angiogenesis, cancer dormancy, anti-inflammation, mineralization (Azoidis et al., 2018; Casson et al., 2018; Baruah and Wary, 2019; Zhang H. et al., 2019). It is also known that physiologically native EVs actively interact with the ECM (Buzas et al., 2018) and the EV-ECM complexes are mediating significant biological functions of both ECMs and EVs (Sung et al., 2015). Recently, matrix-bound vesicles (MBVs) are identified to play a significant role in mediating the regenerative functions of ECM scaffolds (Huleihel et al., 2016, 2017; van der Merwe et al., 2017; Rilla et al., 2019), which highlights the biological functions of the EV-ECM structural complexes. Therefore, designing and constructing biomaterial scaffolds to mimic the structure of the EV-ECM complex and the physiological interactions between the ECM and EVs, represents an attractive and promising novel approach for tissue engineering applications. Mesenchymal stem cells (MSCs) isolated from various tissues are multipotent stem cells (Fridenshtein et al., 1968; Jo et al., 2007; Oh et al., 2008; Zannettino et al., 2008; Xue et al., 2018), represent a promising regenerative treatment for a variety of diseases (Bouffi et al., 2010; Lankford et al., 2015, 2017; Wang et al., 2015; Brown et al., 2016; Hofer and Tuan, 2016; Kabagambe et al., 2017; Galganski et al., 2019; Vanover et al., 2019; Zhang Z. et al., 2019), especially vascular diseases (Pankajakshan and Agrawal, 2014; Premer et al., 2019), due to the biofunctional paracrine secretion, including EVs. However, in many of the cases where therapeutic effects were observed using MSCs, the transplanted stem cells did not persist following injection and thus did not contribute to tissue regeneration by integration. Therefore, MSC derived EVs represent a promising alternative with sustained paracrine functions of live MSCs and have great potential for cell-free therapy. Although the functions of the EVs are not as comprehensive as the MSCs, EVs derived from different types of MSCs also have been demonstrated for improving tissue regeneration (Zhang et al., 2015; Liang et al., 2016; Merino-Gonzalez et al., 2016). Among other molecules, integrins on the surface of EVs are of critical functional significance as they regulate the interactions between EVs and the surrounding micro milieu, especially ECM molecules (Clayton et al., 2004; de Jong et al., 2016; Buzas et al., 2018). Therefore, developing integrin-based conjugation approaches to immobilize MSC-derived EVs onto biomaterial scaffolds will mimic the EV-ECM complexes and hold promise for tissue engineering applications.

One-bead one-compound (OBOC) combinatorial technology is an ultra-high throughput chemical library synthesis and screening method, which is suitable for integrin-based ligand discovery (Lam et al., 1991). Previously, we have identified various potent ligands, such as LXY30, LXW7, and LLP2A targeting integrins α3β1, αvβ3, and α4β1, respectively, by employing the OBOC combinatorial technology (Peng et al., 2006; Xiao et al., 2010, 2016). We also have identified LLP2A had strong binding to human placental chorionic villus MSCs (PMSCs) (Hao et al., 2019) via integrin α4β1 and established an approach to immobilize LLP2A onto electrospun scaffold (Hao et al., 2020b). It has been shown that EVs derived from MSCs, especially from PMSCs, could stimulate angiogenesis (Komaki et al., 2017). Thus, in this study, we propose to immobilize PMSC-derived EVs (PMSC-EVs) onto native ECM mimicking electrospun nanofibrous scaffold by using LLP2A as the conjugation linker to construct the functional biomaterial scaffold to mimic the natural EV-ECM structural complexes and promote the vascularization and regeneration potential for tissue regeneration applications.

## Materials and Methods

### Cell Culture

We used PMSCs isolated from early gestation placental chorionic villus tissue as described in our previous studies (Lankford et al., 2017; Hao et al., 2019; Kumar et al., 2019). PMSCs were expanded in D5 medium containing high-glucose DMEM (HyClone), 5% fetal bovine serum (FBS, HyClone), 20 ng/mL recombinant human basic fibroblast growth factor (bFGF, R&D systems), 20 ng/mL recombinant human epidermal growth factor (EGF, R&D systems), 100 UI/mL of penicillin and 100 μg/mL of streptomycin and incubated at 37°C, 5% CO_2_. PMSCs were used between P3 and P5 for all experiments. Human umbilical vein endothelial cells (HUVECs) were purchased from Lonza and expanded in EGM-2 media (Lonza). HUVECs were used between P3 and P6 for all experiments.

### EV Isolation and Characterization

EV isolation and characterization were performed using an established protocol described in our previous study (Kumar et al., 2019). Briefly, PMSCs were seeded at a density of 20,000 cells/cm^2^ in tissue culture–treated T175 flasks in 20 mL of EV-depleted FBS containing D5 medium for 48 h at 37°C, 5% CO_2_. Conditioned medium was collected and sequentially centrifuged at 300 g for 10 min, 2,000 g for 20 min, and passed through a 0.2 mm filter. Then, the medium was concentrated using Amicon Ultra 15 Centrifugal Filter Units with a 100 kDa MW cutoff (MilliporeSigma), transferred to thickwall polypropylene tubes (Beckman Coulter), and centrifuged at 8,836 g using the SW28 rotor and L7 Ultracentrifuge (Beckman Coulter). The supernatant was transferred to fresh tubes, centrifuged at 112,700 g for 90 min, and the pellet was resuspended in PBS (HyClone) and spun again at the same speed and time. The final pellet was resuspended in 10 mL of PBS per T175 flask and stored in aliquots at −80°C. The number and size distribution of isolated EVs were characterized by nanoparticle tracking analysis (NTA) using the NanoSight LM10 (Malvern Panalytical, Malvern, United Kingdom) equipped with a 404-nm laser and sCMOScamera. The morphology of isolated EVs was characterized by Transmission electron microscopy (TEM) using a CM120 transmission electron microscope (Philips/FEI BioTwin, Amsterdam, Netherlands) at 80 kV. For Western blot analysis, 10 mL of EVs were treated with either NuPAGE LDS Sample Buffer (Thermo Fisher Scientific) containing reducing agent DTT, for detecting ALIX, tumor susceptibility gene 101 (TSG101), calnexin, integrin α4 and integrin β1 proteins, or without DTT, for detecting CD9 and CD63 proteins), and heated to 90°C. The samples were run, transferred, probed with primary antibodies ALIX, TSG101, CD9 (MilliporeSigma), calnexin (Cell Signaling Technology), CD63 (Thermo Fisher Scientific), Integrin α4 (MilliporeSigma) and Integrin β1 (MilliporeSigma) at 4°C overnight. Subsequently, membranes were incubated for 1 h with conjugated secondary antibodies (Cell Signaling Technology) at room temperature and blots were imaged using a ChemiDoc MP^+^ imaging system (Bio-Rad).

### Cell Migration Assay

HUVECs were seeded in Culture-Insert 2 Well in μ-Dish (ibidi) and cultured in EBM-2 with 1% BSA (Thermo Fisher Scientific) with EVs or without EVs and incubated at 37°C, 5% CO_2_ for 12 h. Images were taken using a Carl Zeiss Axio Observer D1 inverted microscope. The cell migration area was quantified using ImageJ software (NIH).

### Rat Aortic Ring Assay

All procedures were approved by the Institutional Animal Care and Use Committee at the University of California, Davis. Ten-week-old male Sprague Dawley rats were purchased from the Charles River animal facility. Rat abdominal aortas were dissected and sliced into 1 mm sections. Aortic rings were embedded in Matrigel® Growth Factor Reduced (GFR) Basement Membrane Matrix (Corning) and cultured in EBM-2 medium with 1% BSA with or without EVs and incubated at 37°C, 5% CO_2_. Angiogenic sprouts were counted after 7 days of culture. The number of sprouts was quantified using Wimasis Image Analysis.

### Exo-Glow Labeling of EVs

EVs were labeled using Exo-Glow (Green) (System Biosciences) according to manufacturer's instructions. Briefly, Exo-Glow (Green) was diluted 1:500 in PBS and added to EVs. The sample was mixed gently by flicking the tube and incubated at 37°C for 10 min. Twenty microliters of ExoQuick-TC (System Biosciences) were added to stop the labeling reaction and the sample was incubated on ice for 30 min. Excess dye was removed by centrifuging at 16,000 g for 3 min at 4°C. The EV pellet was resuspended in 500 μL of cold PBS, placed on ice for 5 min, and centrifuged again at 16,000 g for 3 min at 4°C. Washes were repeated for a total of 3 times. After the final wash, labeled EVs were resuspended in 500 μL of PBS. As a control, the same process was repeated using with same volume of PBS that the EVs were re-suspended in.

### EV Attachment on LLP2A Treated Surface

To modify the culture surface with ligands, cell culture wells in a 48-well plates were coated with 150 μL of 20 μg/mL Avidin (Thermo Fisher Scientific) and incubated for 1 h at 37°C. Avidin coated wells were rinsed three times with PBS and were treated with 150 μL molar equivalents (2 μM) of D-biotin (Thermo Fisher Scientific) or LLP2A-bio. After 1 h, the wells were washed three times with PBS and blocked with 1% BSA for 1 h. After the wells were rinsed three times with PBS, for the EV attachment assay, 5 × 10^6^ EVs suspended in PBS was added into each well and incubated for 10 min at 37°C and 5% CO_2_. Then the wells were washed three times with PBS, and the adhered EVs were imaged using a Carl Zeiss Axio Observer D1 inverted microscope. Quantification of images was performed using the ImageJ software.

### Quantitative Reverse Transcription Polymerase Chain Reaction (qRT-PCR) Assay and Caspase 3 Assay

For the treated culture surface assay, three different groups, untreated, LLP2A treated and EV treated were set up in 24-well plates. As described above, the untreated wells were coated with D-bio, the LLP2A treated wells were coated with LLP2A and the EV treated wells were seeded with EVs on the LLP2A treated surface. HUVECs were seeded in wells with different treatments and cultured in EBM-2 media with 1% BSA at 37°C and 5% CO_2_ for 48 h. RNA extraction from cells was performed using RNeasy Mini Kit (Qiagen) according to the manufacturer's instructions, and cDNA was synthesized using Superscript II Reverse transcriptase (ThermoFisher Scientific). PCR was performed using the Biorad CFX96 Real-Time PCR Detection System (BioRad Laboratories) machinewith the SsoAdvanced SYBR Green Supermix (Bio-Rad). Amplification conditions after an initial denaturation step for 90 s at 95°C were 40 cycles of 95°C, 10 s, for denaturation, 55°C, 10 s, for annealing and 72°C, 30 s, for elongation. GAPDH was used as the reference gene for calculations. Data were analyzed by the 2DDCT threshold cycle method. Primer sequences are listed in Table 1. For caspase 3 assay, to mimic the ischemic environment when ECs often experience after implantation *in vivo* into the wounded or defect area, EC survival was characterized in a hypoxic chamber as described previously (Hao et al., 2019). HUVECs were seeded on different treated surfaces and cultured in hypoxia chamber in EBM-2 media at 37°C, 1% O_2_ and 5% CO_2_ for 6 h, and then lysed and analyzed by using a caspase 3 Assay Kit (Cell Signaling Technology) according to the manufacturer's instruction. Fluorescence (ex 380 nm/em 450 nm) was measured using a SpectraMax i3x Multi-Mode Detection Platform (Molecular Devices).

**Table 1:** Primers used for qRT-PCR.

**Gene**	**Forward primer**	**Reverse primer**
KDR	CCAAGAACTCCATGCCCCTTA	ATCCCTGGGATCTGAAACG
TIE2	TAGAGCCTGAAACAGCATACCAGG	CTATTGGAATGGCAAATGCTGGG
GAPDH	ACCACAGTCCATGCCATCAC	TCCACCACCCTGTTGCTGTA

### Preparation of EV-Modified Electrospun Scaffolds

Preparation of EV-modified electrospun scaffolds included three steps as shown in **Figure 5**: (1) construction of electrospun scaffolds using the established electrospinning technology, (2) LLP2A immobilization on the electrospun scaffolds via “Click chemistry,” and (3) EV immobilization on the LLP2A modified electrospun scaffolds via integrin-based conjugation. Construction of electrospun scaffolds was performed as previously reported (Hao et al., 2020b). Briefly, The polymer blends (e.g., 19% PLLA and 5% PCL; w/v) were completely dissolved in 1,1,1,3,3,3-hexafluoro-2-propanol (HFIP, Aladdin). Electrospun scaffolds were prepared by electrospinning polymer fibers onto the rotating drum collector. A negative voltage of 4.5 kV was applied to the mandrel, and a positive voltage of 4 kV was applied to the spinneret, by using a high voltage generator (Gamma High Voltage). LLP2A was grafted onto the PLLA/PCL membrane surface as our previously reported in three steps (Hao et al., 2020b). First, scaffolds were incubated in 0.01 M sodium hydroxide for 10 min to expose the carboxyl groups on the surface. Second, the scaffolds were further incubated in a solution of 1-ethyl-3-(3-(dimethylamino)propyl)- carbodiimide hydrochloride (EDC, Thermo Fisher Scientific) and N-hydroxysulfosuccinimide (sulfo-NHS, Thermo Fisher Scientific) in 0.5 M morpholino ethanesulfonic acid (MES) buffer (Thermo Fisher Scientific) for 30 min. After washing with PBS, the scaffolds were incubated in a solution of azido-PEG11-amine (N_3_-PEG11-NH_2_, BroadPharm) in alkalescent PBS (pH = 7.8) for 2 h on a shaker. Third, 20 mM LLP2A-DBCO was conjugated to azido-decorated scaffolds via “Click chemistry” in water for 16 h.

To evaluate the EV immobilization on the LLP2A modified electrospun scaffolds, EVs were seeded on electrospun scaffolds modified with different density of LLP2A (0, 50% or 100%). Briefly, we used another peptide ligand LXW7, specifically binds to αvβ3 integrin, but not integrin α4β1, described in our previous studies (Hao et al., 2017, 2020a) as the competitor ligand to LLP2A to modify the electrospun scaffolds. We used three conditions with different molar ratios of LLP2A-DBCO/LXW7-DBCO to modify the scaffolds: (1) PBS, (2) solution with equal molar ratio of LLP2A-DBCO/LXW7-DBCO (0.5:0.5), and (3) solution with LLP2A only (LLP2A-DBCO/LXW7-DBCO=1:0) to represent 0, 50% density of LLP2A, and 100% density of LLP2A. The scaffolds were washed with PBS for three times. The EV immobilization was characterized by using Scanning Electron Microscope (SEM, Hitachi TM-1000). Quantification of images was performed using the ImageJ software.

### Evaluation of Angiogenic Gene Expression, Apoptosis and Survival of HUVECs

For angiogenic gene expression, HUVECs were seeded on electrospun scaffolds modified with or without EVs and cultured in EGM-2 medium at 37°C, 20% O_2_ and 5% CO_2_ for 48 h, and the gene expression was evaluated by qRT-PCR as described above. For apoptosis and survival assay, the ischemic environment was set up as described above. HUVECs were seeded on electrospun scaffolds modified with or without EVs and cultured in hypoxia chamber in EBM-2 medium at 37°C, 1% O_2_ and 5% CO_2_. For Annexin V staining, the cells were cultured for 6 h, the staining was performed by using the Annexin V-FITC antibody (Abcam) according to the manufacturer's instructions. The images were collected using a Carl Zeiss Axio Observer D1 inverted microscope. Quantification of images was performed using the ImageJ software. For caspase 9 assay and caspase 3 assay, the cells were cultured for 6 h, and then lysed and analyzed by using a caspase-9 Activity Assay Kit (Abcam) or the caspase 3 Assay Kit, respectively, according to the manufacturer's instructions. Fluorescence (ex 380 nm/em 450 nm) was measured using a SpectraMax i3x Multi-Mode Detection Platform. For cell survival assay, the cells were cultured for 4 days and then determined using the MTS assay according to the manufacturer's instruction. The amount of soluble formazan product produced by the reduction of MTS by metabolically active cells was measured at the 490 nm absorbance using the SpectraMax i3x Multi-Mode Detection Platform.

### Statistical Analysis

For two-sample comparison, a student's *t*-test was used. For multiple-sample comparison, analysis of variance (ANOVA) was performed to detect whether a significant difference existed between groups with different treatments. A p-value of 0.05 or less indicates a significant difference between samples in comparison.

## Results and Discussion

### Characterization of PMSC-EVs

**Figure 1:**
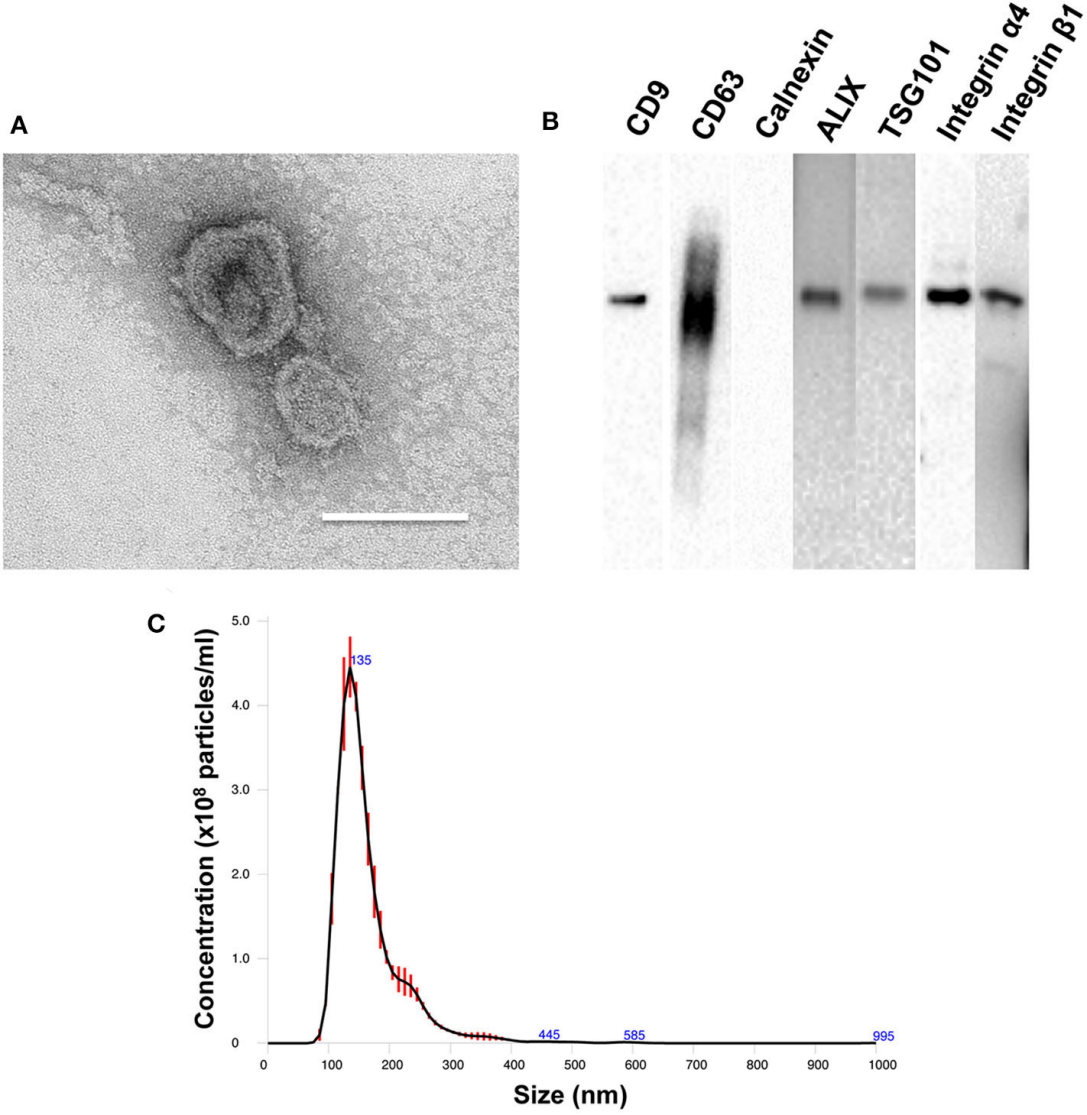
Characterization of PMSC-EVs. **(A)** TEM image of PMSC-EVs. Scale bar = 150 nm. **(B)** Western blot analysis of CD9, CD63, calnexin, ALIX, TSG101, integrin α4 and integrin β1 of PMSC-EVs. **(C)** NTA analysis of PMSC-EVs.

TEM analysis showed the characteristic cup shape and size of PMSC-EVs (Figure 1A). NTA showed that PMSC-EVs have a size range of 137.4 ± 3.6 nm, which is within the expected size range of EVs (Figure 1C). Western blot analysis confirmed the presence of characteristic EV markers CD9, CD63, ALIX and TSG101, and the absence of endoplasmic reticulum marker calnexin (Figure 1B). The mechanism of EV binding to natural ECM is important to explore the applications of EVs in tissue regeneration, especially to achieve the conjugation of EVs to biomaterials for modification (Huang et al., 2016). EVs adhere to ECM constituents in the integrin dependent manner (Clayton et al., 2004). Thus, the integrin expression on PMSC-EVs was also determined. Our previous study showed that PMSCs highly expressed integrin α4 and integrin β1 (Hao et al., 2020b). Also, EVs carry markers of their original cells (van der Pol et al., 2012). Thus, in addition to the characteristic EV markers, the Western blot results also confirmed that PMSC-EVs expressed integrin α4 and integrin β1 that could be used as the junction to conjugate PMSC-EVs to biomaterial for simulating the natural ECM. The integrin α4 and integrin β1 expression results were also consistent with the report related to protein identification of EVs in our previous study (Kumar et al., 2019).

### PMSC-EVs Promoted EC Migration and Sprouting

**Figure 2:**
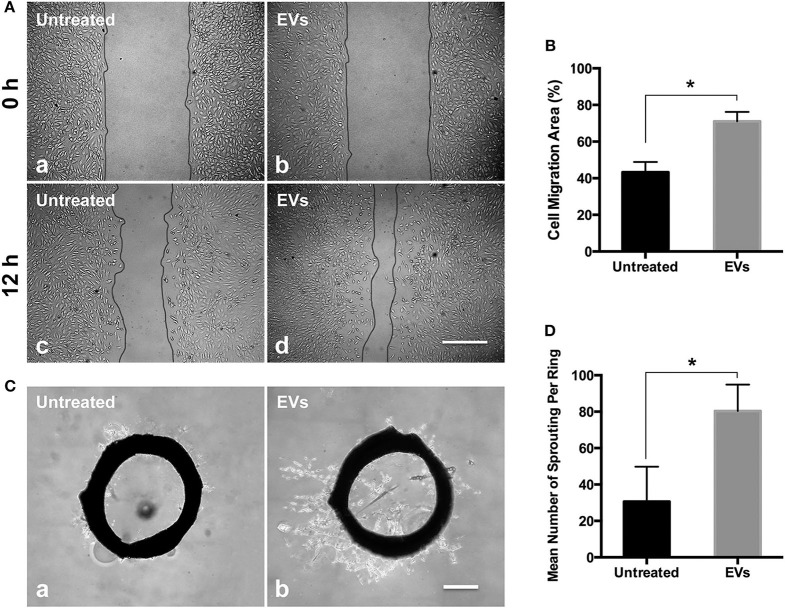
Proangiogenic capacities of PMSC-EVs. **(A)** HUVEC migration treated with PMSC-EVs (b, d) or without PMSC-EVs (a, c). Scale bar = 100 μm. **(B)** Quantification of HUVEC migration area. Data were expressed as mean ± standard deviation: *p < 0.05 (*n* = 3). **(C)** Rat aortic ring assay treated with PMSC-EVs (b) or without PMSC-EVs (a). Scale bar = 500 μm. **(D)** Quantification of the number of sprouting per ring. Data were expressed as mean ± standard deviation: *p < 0.05 (*n* = 3).

Angiogenesis is the crucial need for tissue regeneration (Bi et al., 2012). MSC derived EVs have already developed for the new impetus for improving angiogenesis in tissue regeneration, and EVs derived from different types of MSCs have been demonstrated promoted angiogenesis (Shi et al., 2019). The EC migration results showed that PMSC-EVs significantly enhanced the migration of HUVECs compared to the control group (Figures 2A,B). The rat aortic ring assay is an *ex vivo* model of angiogenesis that studies the effects of mediators on normal vessel sprouting, which showed that PMSC-EVs significantly promoted vessel sprouting compared to the control group (Figures 2C,D). These results indicate that PMSC-EVs prossess strong proangiogenic capacitiy that will extend the PMSC-EV applications in tissue regeneration.

### LLP2A Treated Surface Improved the Attachment of PMSC-EVs

**Figure 3:**
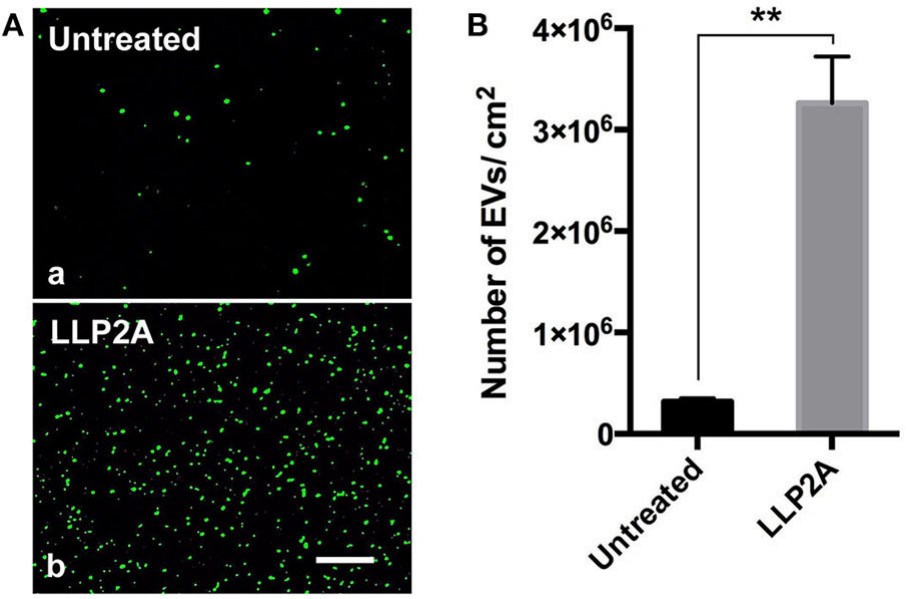
Attachment of PMSC-EVs on different treated surfaces. **(A)** Images of attached PMSC-EVs on untreated surface (a) and LLP2A treated surface (b). Scale bar = 5 μm. **(B)** Quantification of the numbers of PMSC-EVs attached on different surfaces. Data were expressed as mean ± standard deviation: **p < 0.01 (*n* = 5).

The addition of tool molecules, such as biotin, to ligands can be advantageous when used in combination with other components and when used to expand the bioengineering applications of the ligands (Hao et al., 2020b). Our previous work has shown that biotinylation of the ligand did not decrease its binding affinity and showed nearly identical binding strength to the targeted integrin (Hao et al., 2017). Therefore, we conjugated LLP2A to biotin (LLP2A-bio), as described in our previous study (Peng et al., 2006). We used LLP2A-bio or D-biotin (untreated, as control) to treat the culture surfaces and investigated the attachment of PMSC-EVs on different culture surfaces. Before the PMSC-EV seeding, the PMSC-EVs were labeled with Exo-Glow (green) to facilitate the imaging. The results showed that only a few PMSC-EVs attached on the untreated surface (Figure 3A, a) and a number of PMSC-EVs attached on the LLP2A untreated surface (Figure 3A, b). The numbers of attached PMSC-EVs on different treated surfaces were quantified, which showed the LLP2A-treated surface significantly improved PMSC-EV attachment compared to the untreated surface (Figure 3B).

### PMSC-EV Treated Surface Improved Angiogenic Activity and Suppressed Apoptotic Activity of ECs

**Figure 4:**
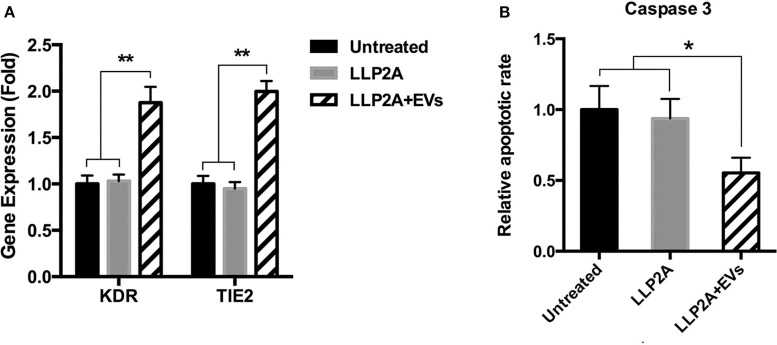
Effects of PMSC-EV treated surface on angiogenic and apoptotic activity of HUVECs. **(A)** KDR and TIE2 expression of HUVECs cultured on untreated surface, LLP2A treated surface or PMSC-EV treated surface. **(B)** Caspase 3 activity of HUVECs cultured on untreated surface, LLP2A treated surface or PMSC-EV treated surface. Data are expressed as mean ± standard deviation: *p < 0.05, **p < 0.01 (*n* = 4).

Angiogenic gene expression, such as KDR and TIE2, is the result of new vessel formation and the improvement of the process of tissue regeneration (Malecki et al., 2005). Upon ligand binding, integrins activate signal transduction pathways that mediate cellular signals (Giancotti and Ruoslahti, 1999). Our previous study has demonstrated that LLP2A was an integrin α4β1 ligand (Hao et al., 2020b). To avoid the effect of LLP2A on the gene and protein expression of cells, we set up LLP2A treated surface as another control group. The results showed that PMSC-EV treated surface significantly increased the expression of the angiogenic genes, KDR and TIE2, compared to untreated surface and LLP2A untreated surface, and no significant difference was shown between untreated surface and LLP2A untreated surface (Figure 4A). Caspase-3 has been found to be necessary in apoptosis due to it is responsible for chromatin condensation and DNA fragmentation (Porter and Janicke, 1999). The results showed that PMSC-EV treated surface significantly decreased the expression of caspase 3 compared to untreated surface and LLP2A untreated surface, and no significant difference was shown between untreated surface and LLP2A untreated surface (Figure 4B). These results indicate that PMSC-EV treated surface is able to improve angiogenic activity and suppressed apoptotic activity of ECs, but LLP2A treated surface does not have impact on angiogenic and apoptotic activity of ECs.

### Preparation and Characterization of PMSC-EV Modified Electrospun Scaffold

**Figure 5:**
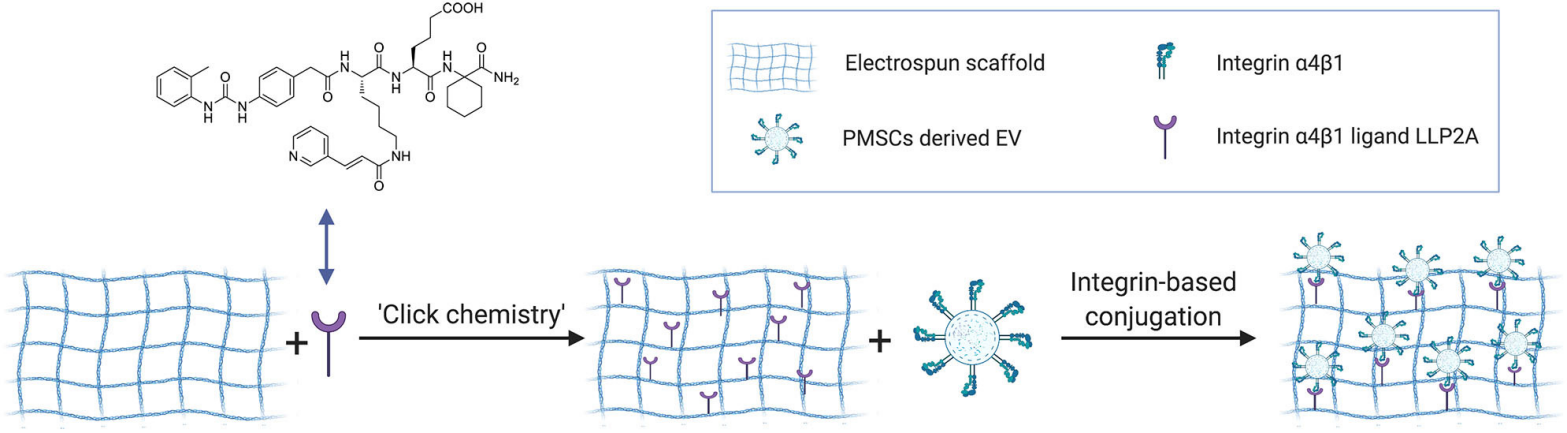
Schematic diagram showing the use of “Click chemistry” to introduce LLP2A to the electrospun scaffold, and the use of immobilized LLP2A to capture PMSC-EVs onto the scaffold surface.

**Figure 6:**
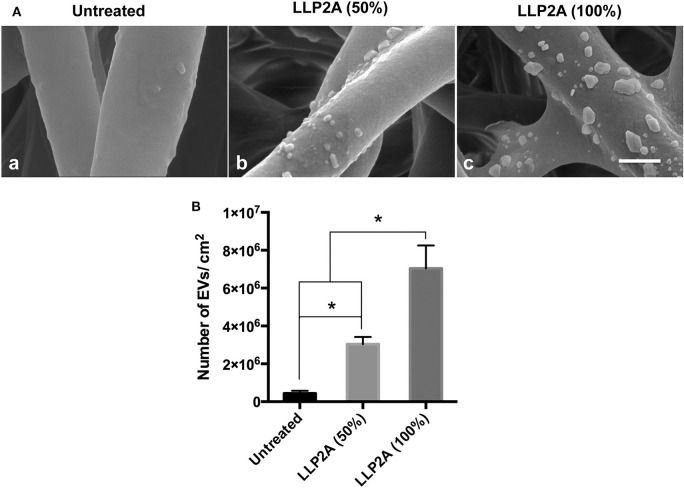
Effect of LLP2A modification on PMSC-EV attachment on electrospun scaffolds. **(A)** SEM images of PMSC-EV attachment on untreated electrospun scaffold (a), 50% LLP2A modified electrospun scaffold (b) and 100% LLP2A modified electrospun scaffold (c). Scale bar = 500 μm. **(B)** Quantification of the numbers of PMSC-EVs on the different modified electrospun scaffolds. Data were expressed as mean ± standard deviation: *p < 0.05 (*n* = 5).

ECM is a three-dimensional (3D) network of extracellular macromolecules and the crucial need to provide structural and biochemical support to surrounding cells (Bonnans et al., 2014; Rabelink et al., 2017). To mimic the natural ECM structure, we employed electrospinning technology to construct network electrospun scaffolds as the description in our previous study (Hao et al., 2020b). To increase the integrin binding sites on the electrospun scaffolds, we developed a protocol to immobilize LLP2A, an integrin α4β1 ligand, onto the electrospun scaffolds via “Click chemistry” as the description in our previous study (Hao et al., 2020b). To mimic the biological EV-ECM complexes of natural ECM, we conjugated the integrin α4β1 expressing PMSC-EVs onto the electrospun scaffolds via integrin-based binding approach (Figure 5). The PMSC-EV modified electrospun scaffold was evaluated using SEM. The results showed the 50% LLP2A modified electrospun scaffolds showed significantly increased immobilization of PMSC-EVs compared to the control untreated electrospun scaffolds, and the 100% LLP2A modified electrospun scaffolds further significantly increased the PMSC-EV immoblization compared to the 50% LLP2A modified electrospun scaffolds (Figure 6A), and the quantification of the numbers of immobilized PMSC-EVs on the electrospun scaffolds showed linear correlation with the densities of LLP2A used indicates that the PMSC-EV immobilization system we developed in this study is mechanistically mediated by the densities of LLP2A molecules (Figure 6B) and the PMSC-EVs have been successfully immobilized on the electrospun scaffold via LLP2A binding approach. The SEM results showed that some EVs seemed aggregated on the ligand modified scaffolds. It is known that EV aggregation could occur during isolation, storage or upon freeze and thaw (Bosch et al., 2016) and the EVs we used in this study were frozen stored before the immobilization process. Therefore, we believe that the aggregation of EVs seen on the scaffolds happened due to the storage of the EVs. To minimize EV aggregation, avoiding the freeze-thaw cycles of EVs and adding Trehalose into the frozen stock solution could prevent aggregation and cryodamage of EVs according to the previous study (Bosch et al., 2016). We do not anticipate that the EV immobilization strategy we developed in this study will facilitate EV aggregation, because in our design, we conjugated EVs onto electrospun scaffolds by using LLP2A as a linker, and LLP2A was first conjugated onto the electrospun scaffold, then the EVs were immobilized onto the LLP2A modified electrospun scaffold. EVs were never in direct contact with free LLP2A molecules, therefore EV aggregation, if any, should not be caused due to their interactions with free LLP2A molecules. In addition, LLP2A is a small peptide molecule that can bind to integrin molecules on the surface of EVs, and one LLP2A molecule that is immobilized to the scaffold surface can only bind to one integrin molecule. Therefore, we do not anticipate the immobilized LLP2A molecules would affect the aggregation of EVs directly.

### PMSC-EV Modified Electrospun Scaffolds Improved the Angiogenic Activity and Survival of ECs

**Figure 7:**
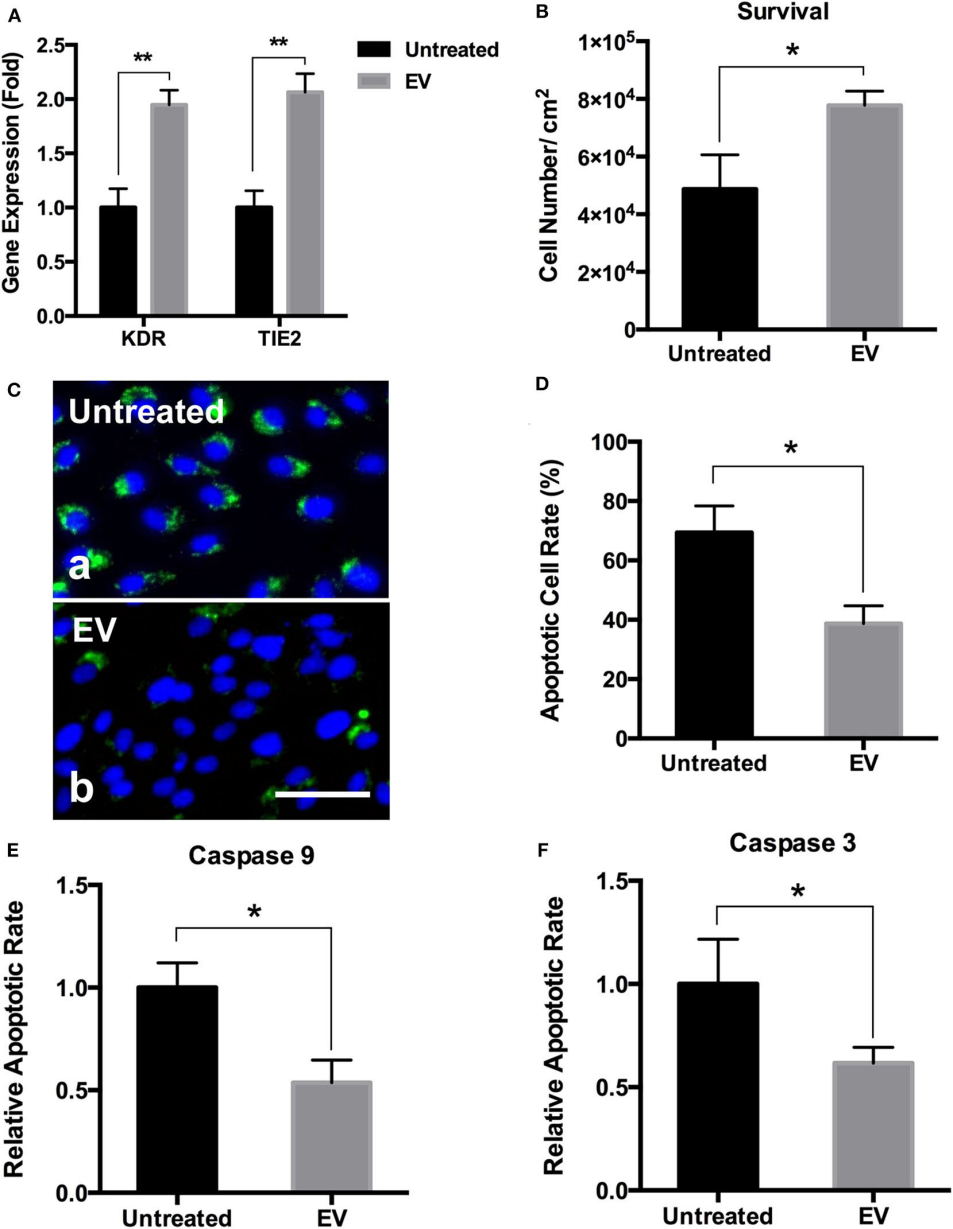
Effects of PMSC-EV modified electrospun scaffolds on angiogenic gene expression and apoptotic rate and protein expression of HUVECs. **(A)** Quantification of KDR and TIE2 expression of HUVECs cultured on untreated electrospun scaffolds and PMSC-EV modified electrospun scaffolds. **(B)** Quantification of HUVEC survival on untreated electrospun scaffolds and PMSC-EV modified electrospun scaffolds. **(C)** Annexin V staining of HUVECs cultured on untreated electrospun scaffolds (a) and PMSC-EV modified electrospun scaffolds (b). **(D)** Quantification of apoptotic rate of HUVECs cultured on untreated electrospun scaffolds and PMSC-EV modified electrospun scaffolds. **(E)** Quantification of caspase 9 expression of HUVECs cultured on untreated electrospun scaffolds and PMSC-EV modified electrospun scaffolds. **(F)** Quantification of caspase 3 expression of HUVECs cultured on untreated electrospun scaffolds and PMSC-EV modified electrospun scaffolds. Data were expressed as mean standard deviation: *p < 0.05, **p < 0.01 (*n* = 4).

Angiogenic gene expression is essential for the regulation of new vessel formation that is fundamental to the development and maintenance of regenerative tissues (Holden and Nair, 2019). The results showed that compared to the untreated electrospun scaffolds, PMSC-EV modified electrospun scaffolds significantly improved the expression of angiogenic genes, KDR and TIE2, of HUVECs (Figure 7A). EC transplantation is an effective approach to improve vascularization and integration of the transplanted biomaterial-based scaffolds, however, EC survival is a significant challenge for the success of cell-based functional biomaterial implants for tissue regeneration (Foster et al., 2018). Apoptosis is a form of programmed cell death that is regulated by the caspase family of proteins, such as caspase 9 and caspase 3, activation of which is the result of intrinsic apoptosis (Hassan and Amer, 2011; Brentnall et al., 2013). Many previous studies have demonstrated that MSC derived EVs prevent cell apoptosis by regulating the activation of caspase 9 and caspase 3 (Li et al., 2019; Liu et al., 2019). In addition, Annexin V is commonly used to detect apoptotic cells by its ability to bind to phosphatidylserine, a marker of apoptosis expressed on the outer leaflet of the plasma membrane. The results showed PMSC-EV modified electrospun scaffolds significantly improved HUVEC survival (Figure 7B), decreased the apoptotic rate (Figures 7C,D) and the expression of caspase 9 and caspase 3 of HUVECs (Figures 7E,F). These results indicate PMSC-EV modified electrospun scaffolds will be worth expecting in vascular tissue regeneration. The main innovation of this study is that it established a novel approach to immobilize the PMSC-EVs onto the electrospun scaffolds by using an integrin-based method that mimics the MBVs in native ECM. This EV delivery system was to modify the polymeric scaffold with EVs to confer the biological functions of EVs to the scaffold. The EVs possess multiple functions, therefore, the EV-modified scaffolds could be widely applied in different areas. Further detailed evaluation of the functions of EV-modified scaffolds in different *in vivo* models is warranted in future studies. The release of the EVs from the scaffold is crucial for the regenerative capacity of this EVs modified scaffold. We anticipate that when used *in vivo*, the PMSC-EVs modified scaffold could transfer the biological information inside the EVs to the cells that are in direct contact with the scaffolds via EV-cell fusion without the need of releasing the EVs to the body system. Meanwhile, immobilized PMSC-EVs on the scaffold could also be released from the scaffold to the body system. The EV delivery system we designed in this study is based on chemical modification of the scaffolds with the N_3_-PEG11-NH_2_ linker and DBCO-LLP2A via “Click chemistry” and then the molecular interaction mediated by LLP2A and integrin α4β1 on the surface of EVs. Since the LLP2A-integrin binding is an “on-and-off” non-covalent interaction, therefore, we anticipate PMSC-EVs will be released first off from the scaffold by the dissociation of the LLP2A from the binding pocket of integrin α4β1. In the long-term, PMSC-EVs will be released from the scaffold because of the break of the covalent bonds between LLP2A and electrospun scaffold as well as the biodegradation of the electrospun scaffold.

## Conclusion

In this study, we isolated EVs from PMSCs and demonstrated the PMSC-EVs possessed pro-angiogenic capacity and anti-apoptotic capacity. We successfully established an integrin-based binding technology to immobilize the PMSC-EVs onto the electrospun ECM-mimicking scaffolds to mimic the EV-ECM complexes. The PMSC-EV modified electrospun scaffolds promoted EC angiogenesis and prevented EC apoptosis in ischemic environment. This study demonstrates that EV modified biomaterials represent a new functional biomaterial and hold promise for tissue engineering and regenerative medicine applications.

## Data Availability Statement

All datasets generated for this study are included in the article material.

## Ethics Statement

The animal study was reviewed and approved by institutional animal care and use committee at the University of California, Davis.

## Author Contributions

DH performed the evaluation of PMSC-EVs, performed the construction and evaluation of the PMSC-EVs modified electrospun ECM-mimicking scaffolds, wrote the manuscript, and discussed the results. DH and HS performed the isolation and characterization of the PMSC-EVs. LR performed the labeling of PMSC-EVs. KL and RL performed the chemical synthesis and discussed the results. DF discussed the results. AW was responsible for conceptualization, results discussion revising the manuscript. All authors contributed to the article and approved the submitted version.

## Conflict of Interest

The authors declare that the research was conducted in the absence of any commercial or financial relationships that could be construed as a potential conflict of interest.
